# Enhancing educational technology practical course: Designing and validating tools for evaluating knowledge, performance, and satisfaction of public health students

**DOI:** 10.1002/hsr2.2108

**Published:** 2024-05-21

**Authors:** Fatemeh Darabi, Zahra Karimian

**Affiliations:** ^1^ Department of Public Health Asadabad School of Medical Sciences Asadabad Iran; ^2^ Department of Medical Education Smart University of Medical Sciences Tehran Iran; ^3^ Department of e‐Learning in Medical Sciences, Virtual School and Center of Excellence in e‐Learning Shiraz University of Medical Sciences Shiraz Iran

**Keywords:** educational material, educational technology, health education, knowledge, performance, questionnaire validation, satisfaction

## Abstract

**Background:**

The present study was conducted with the aim of designing and validating questionnaires for evaluating the public health students' knowledge, performance, and satisfaction (KPS) in the course of educational technology.

**Methods:**

The present study was conducted with qualitative‐quantitative approach. Qualitative stage was done based on experts' opinion and related articles and questionnaires for developing, designing, and validating the tools. For validating the tools, face validity and content validity was done based on the opinion of the 10 expert panel. In quantitative stage, all initial validated tools were implemented on 50 students of public health for determining the reliability. The reliability of the tools was calculated using the internal consistency method of the items with Cronbach's *⍺* coefficient, and Kuder–Richardson. All analyses were conducted using SPSS‐19 software.

**Results:**

In the field of qualitative research, an assessment tool consisting of 20 four‐option questions was designed. The content validity of this tool was confirmed based on the opinions of 10 educational experts, with CVI Total = 0.862 and CVR = 0.885. Additionally, the reliability of the tool was validated through testing on 50 public health students, resulting in a reliability coefficient of *r* = 0.780. Furthermore, for performance evaluation, four assessment tools each comprising 20 questions (totaling 80 questions) were designed. The validity values for these tools in the areas of PowerPoint (CVI = 0.981, CVR = 0.960), pamphlet (CVI = 0.866, CVR = 0.877), poster (CVI = 0.871, CVR = 0.906), role‐playing (CVI = 0.871, CVR = 0.980), and the reliability of the tools with Cronbach's *⍺ r* > 0.80 were confirmed. In the satisfaction assessment section, a researcher‐made questionnaire consisting of 18 questions across seven domains was designed, with confirmed content validity (CVI = 0.949, CVR = 0.861) and reliability (*r* = 0.928).

**Conclusions:**

It seems that this tool can be used to measure the KPS of students from the educational technology course of the health field and similar fields.

## 1 INTRODUCTION

Universities, being a prominent academic institutes for investing in students as human capital, primarily focus on training and cultivating an effective workforce.^1^ Development of students in the health sector aims to improve service quality, necessitating the efficient training of students.^2^ One of the primary objectives in health and medical education should focus on instructing and preparing students in professional skills and personal growth, enhancing their self‐directed learning, and fostering social competencies.^3^ Ensuring the quality of students' performance in order to prevent the wastage of human and material capital and to have the ability to compete in the future world, where quality is the most important component for the survival of any organization, is an undeniable necessity.^4^, ^5^ The most important goal of education in the fields of health professions is to develop and create qualifications, to train successful graduates who are capable of using what they have learned.^6^ Health and medical sciences encompass not only the imparting of theoretical knowledge but also the essential task of cultivating practical skills within the field. Public health, as a branch of medical sciences, is particularly instrumental in equipping students with a wide range of practical skills and competencies.^6^ Educational settings for practical training may not always provide ideal learning conditions. However, instructors have the opportunity to enhance the learning environment and maximize the benefits of education by creating favorable conditions, employing diverse strategies, and utilizing various teaching approaches.^7^, ^8^

For this purpose, health science must integrate theoretical and practical knowledge of health based on logic derived from what is already known and what is gained from practical experience. This requires creating practical fields to promote the development of wise, critical, and thoughtful professionals.^9^ The practical learning process should enable students to develop the learned theoretical parts that integrate the application of knowledge with skills and attitudes in real‐life practical situations.^10^

Accordingly, one of the effective criteria of practical education is empowering students, followed by developing knowledge, attitude, and skills. These three components are of significant importance in complex learning and professional roles. Therefore, education can create necessary and needed competencies.^11^, ^12^ Research indicates that designing integrated theoretical and practical activities based on real‐world tasks leads to more effective education for students. By aligning educational tasks with real‐world scenarios, educators can enhance the learning experience and improve educational outcomes.^12^, ^13^, ^14^

Among the fields of medical sciences is public health, where effective education in the dimensions of knowledge, attitude, and skills in these fields is of particular importance and should be paid attention to from the beginning of entering the university and in basic courses.

The educational technology course is one of the basic theoretical‐practical subject that are offered to learn the practical skills of the students of this field, and it has a special place in the skills training of the learners and will play an important role in their job performance.^15^

Educational technology plays a crucial role in enhancing students' personality growth and motivation for learning.^16^ Studies^17^, ^18^ have highlighted the significance of innovative education in improving the readiness for learning. The application of educational technology, along with changes and innovative approaches, directly impacts the activities of both professors and students. Introducing innovations into the educational system through updates and changes is essential for the development of education. These innovations positively influence educational activities and contribute to overall improvement in the educational sector.^19^

The course of educational technology and educational aids, in terms of combining theory and practice, makes learning lasting and diversifying in the classroom. In fact, educational technology has played an effective role in improving the efficiency and quality of the teaching and learning process and the academic progress of public health students,^20^ which increases the teaching and learning process of students by using media and educational aids.^21^ Various studies emphasize the positive effects of the potential power of educational technology on improving students' attitudes and performance.^22^

Increased implementation of technology will increase students' comprehension of content and development of skills in such areas as analytical reasoning, problem solving, information evaluation, and creative thinking.^23^ One of the main approaches in health and medical education is the six‐faceted SPICES model, which was presented by Harden with an analysis of the changing process and needs of modern health and medical sciences schools.^24^ The SPICES model in medical education focuses on key elements like student‐centered learning, problem‐based learning, integrated or interprofessional teaching, community‐based education, elective studies, and a systematic approach. This model aims to enhance teaching by promoting student‐centered approaches, problem‐solving skills, and interdisciplinary collaboration.^25^ One of the components of the SPICES model in medical education, particularly relevant in academic and field settings, is the emphasis on systematic training over opportunity‐based training. This aspect highlights the importance of structured and methodical approaches to training, promoting integrative and problem‐solving education within medical contexts.^26^

In the context of health education, it is essential to develop organized and purposeful tools to assess students' knowledge, performance, and satisfaction (KPS). In addition to measuring knowledge and performance, measuring student satisfaction is a crucial aspect of evaluating educational programs, including factors like faculty credentials, financial performance, learner outcomes, comparison with other programs, benchmarking, questionnaires, and hybrid models combining satisfaction and facilities assessment.^27^

Feedback from students plays a vital role in improving medical education programs, helping to identify areas for corrective measures and quality assurance.^28^ Various studies have highlighted the significance of student satisfaction in health education, emphasizing dimensions such as the development of professional competencies, achievement of student expectations, virtual assistance, flexibility in learning, and infrastructure utilization.^29^, ^30^, ^31^, ^32^

To comprehensively evaluate students' KPS in medical education, it is crucial to utilize accurate and reliable tools that cover all dimensions of the course. Various methodologies and tools have been developed to measure student satisfaction in higher education, focusing on aspects like teaching quality, assessment methods, generic skills, learning experiences, and overall student contentment.^33^, ^34^

In Asadabad College of Health Sciences, one of the practical courses along with the public health students' homework is the educational technology course. This course routinely starts with an overview of the theoretical material, and then the students learn from the department's experts, other peers, and self‐study in the educational field. But there were no clear indicators to measure their effective learning criteria. This issue is especially important because the educational technology course is one of the courses, which is important in both theoretical and practical aspects for public health students.

Most of the studies conducted in relation to the validation of students' academic satisfaction scale by Turkzadeh et al.^35^ the construction and validation of the student evaluation questionnaire of educational quality by Rezaei et al., the study of the validation of the Lent academic satisfaction scale in Iranian students by Setayeshi Azhari et al.^36^ no reliable tool was found in this field.

Therefore, according to the importance of the performance and satisfaction of students and according to the experience of the researchers in presenting the practical part of the educational technology course and the students' opinions on how to teach and evaluate this unit; This study was conducted with the aim of designing and validating questionnaires of KPS of public health students in the course of educational technology.

## 2 MATERIALS AND METHODS

### 2.1 Study design

This research was carried out in two stages, qualitative and then quantitative.

#### 2.1.1 Qualitative stage

In the initial stage, the items of the questionnaires for measuring knowledge, satisfaction, and performance were designed using the review of texts, scientific and library resources, similar articles and interviews with three experts in the fields of medical education and health education and health promotion. In this study, 10 experts were selected to check face and content validity. Given that the questionnaires and checklists were researcher‐made and tailored to the educational needs of the target group, experts in the areas of health education (3), medical education (3), and educational technology (4) were utilized to selecting the items and assess the content validity. Also, to determine the face validity of the initial questionnaire, the opinions of the students who had already completed this course were used. Also, to determine the face validity of the questionnaire, the opinions of the students who had already completed this course were used.

#### 2.1.2 Quantitative stage

In the quantitative stage to determine the reliability of KPS, three tools were implemented on all 50 public health students who had taken the course of educational technology in health. To determine reliability, the internal consistency of items with Cronbach's *⍺* coefficient and Kuder–Richardson's method was used. It should be noted that each semester, 25 students are enrolled in this course. To assess the questionnaire's reliability, we involved two cohorts who had completed the course in consecutive semesters. However, it is evident that this study needs replication and examination with a more extensive sample size.

### 2.2 Sampling and participants

To determine the items of assessment tools (checklists and questionnaires) in the field of educational technology, a process was undertaken involving the identification of scientific sources and reputable references in the educational technology course. Subsequently, to establish the content validity of these tools, 10 experts specializing in medical education (3), health education (3), and educational technology (4) were consulted with purposive sampling method. The criteria for expert selection included having a relevant academic background and a minimum of 3 years of teaching experience in the educational technology course. Individuals willing to participate were included in the research, with the option to withdraw if they chose not to continue collaborating.

For the quantitative section and testing of the tools, Undergraduate students majoring in public health from Asadabad medical college who had taken the educational technology course in 2022 were selected. A practical session of the educational technology course was conducted with a total of 50 participants selected. The criteria for student selection included enrollment in the educational technology course, no prior completion of this course, or participation in related open courses on educational technology. Similar to the expert selection process, students willing to engage in the research could opt‐out if they decided not to continue participation.

### 2.3 Educational content and duration

The duration of the educational technology course for public health students was one academic semester, and two consecutive courses for students in 2022 were selected to calculate the reliability of the tool. The educational technology course was presented in two parts: theoretical content presentation (short lecture) and the main practical part (individual and work group activities) in the field of designing and developing the PowerPoint, pamphlets, posters, and role‐playing (Table 1).

**Table 1:** Features of the KPS assessment tool of research.

Tools		Components	*N* of items	Score range	Total scoring scale
Knowledge (MCQ Test) 20 Questions		*Principles of*:			
		Nonreflective educational media	7	0–7	Minimum level = 0 Maximum level = 20 Cut‐off‐point = 10 Minimum good passing = 14
		Reflective educational media	7	0–7	
		Communication	2	0–2	
		Role‐playing	4	0–4	
Performance (4 Checklists) 80 Items		*Practical work of*:			
		Practical work of poster design	20	0–20	Minimum level = 0 Maximum level = 20 Cut‐off‐point = 10
		Pamphlet design	20	0–20	
		PowerPoint design	20	0–20	
		Role‐playing	20	0–20	
Satisfaction (Questionnaire) 18 Items		*Subcomponents*:			
		Motivation	2	1–5	Minimum level = 1 Maximum level = 5 Cut‐off‐point = 3.5
		Learning	3	1–5	
		Problem solving	3	1–5	
		Team working	3	1–5	
		Interaction	3	1–5	
		Time Management	2	1–5	
		Application	2	1–5	
Total			118	‐	

### 2.4 Tools/Instruments

In this study, three different tools were used to measure knowledge, performance, and students satisfaction. The first tool was a 20‐item multiple‐choice questionnaire used to assess students' knowledge. The second tool consisted of four 20‐item checklists to measure students' performance in practical activities, including producing Posters, Pamphlets, PowerPoints, and Role‐playing. Overall, 118 items were used to measure theoretical and practical activities as well as student opinions in this study.

#### 2.4.1 Knowledge assessment questionnaire

This instrument included 20 four‐choice questions in four areas; The principles of Nonreflective educational media, including (posters, pamphlets, Replica, etc. (seven questions), Reflective or fixed transparent educational media educational media (slides, APK, Overhead, PowerPoint, and so forth (seven questions), principles of the communication process to establish education (two questions), and the principles of role‐playing (four questions) scoring scale. The content of the items were extracted from Suresh^37^ Dornan^38^ the source book.

In the check of face validity, which is an initial indicator for checking content validity, the questionnaire was given to 10 experts and they were asked to express their opinions about the questionnaire and its external features (Table 1). At first, the content coverage of items was confirmed by researchers and teacher of the course based on the Learning Objective‐content table and using the opinions of 10. Also, Milman's checklist was used in designing the Multiple‐Choice Questions (MCQ). Then content validity was approved by 10 experts.

#### 2.4.2 Performance measurement questionnaire

In compiling the performance tool using reliable articles and sources and experts' opinion, an instrument consisting of 80 questions in four checklists (20 questions in each checklists) including; Posters, Pamphlets, Role plays, and slides prepared in PowerPoint software were designed. Subscales of poster and pamphlet items included four components: Configuration, Perspective, Content, and Visual elements. The subscales of role‐playing checklists included the three components: Role mastery, Design and Implementation, and control of conditions. The subscales of PowerPoint checklists included five components: Configuration, arrangement of content, scientific content, adjusted content, and Emphasis and attention were compiled. The answers to the performance questions were measured based on the scale of yes with 1 score, no with 0 score, and relatively or somewhat with 0.5 score. In total, the range of scores is between 0 and 40 (considering that the questions of each subscale were 20 questions and the score is between 0 and 1, so the score range is between 0 and 40), finally, the accepted cut‐of‐point was 20 (Table 1).

#### 2.4.3 Satisfaction assessment

This research‐made questionnaire was prepared by studying the related articles in the field of research. This tool consists of 18 questions in seven components included: Motivation (2 Items), Learning (3 Items), Problem solving (3 Items), Team working (3 Items), Interaction (3 Items), Time management (2 Items), and Application (2 Items) was measured with a 5‐point Likert scoring scale (very high, high, medium, low and very low) with a score range of 1–5. The minimum score was 1 and the maximum score was 5, and was acceptable cut‐off‐point score = 3 (Table 1).

### 2.5 Validity and reliability of tools

#### 2.5.1 Face validity index

To determine the face validity of the questionnaires, a complete list of compiled items was given to 10 experts and including health education, medical education, educational technology; to comment on the questions. Also, after developing the initial version and before implementing it on the students, five public health students who had completed this course the previous year confirmed the face validity of the tools.

#### 2.5.2 Content validity

*Content Validity Ratio (CVR)*: In determining the content validity index, 10 experts were asked to comment on the importance and necessity of all questionnaire items. In this approach, each item was examined based on the three‐part spectrum: it is necessary, it is useful but it is not necessary, and it is not necessary. After collecting comments to evaluate the content validity index, CVR was calculated for each question according to the following formula; where *Ne* is the number of experts who consider an item necessary and *N* is the total number of experts. After the calculation, the result was compared with the entry criteria in the Lawshe table according to the number of experts (acceptable 0.62).^39^

$$CVR=\frac{Ne-\frac{N}{2}}{\frac{n}{2}}.$$

2.5.3

*Content validity index (CVI)*: The CVI is the degree of agreement between experts about the relevance, simplicity, and clarity of each item. That is, the number of experts who gave each item a score of 3 and 4 divided by the total number of experts, which is actually the ratio of agreement on the three mentioned indexes of each item. In this method, questions with a score higher than 0.79 are suitable, between 0.70 and 0.79 need to be modified, and less than 0.70 are unacceptable and should be removed.^40^

$$\text{CVI}\;=\frac{\text{Number}\;\text{of}\;\text{raters}\;\text{giving}\;a\;\text{rating}\;\text{of}\;3\;\text{or}\;4}{\text{Total}\;\text{number}\;\text{of}\;\text{raters}}.$$

For knowledge assessment, the reliability of the questions was done using Kuder–Richardson's method. This formula is a special case of Cronbach's *⍺* test which is calculated for dichotomous scores. A high coefficient of KR‐20, for example, more than 0.90 indicates a homogeneous test. The values of this formula can be from 0.00 to 1 (which is sometimes expressed as 0 to 100). The formula used in this method is given below.

$$\alpha =\frac{k}{k-1}\left(1-\frac{\sum_{i=1}^{k}\sigma_{y}^{2}}{\sigma_{x}^{2}}\right).$$

To determine reliability of satisfaction and performance tools, the internal consistency method of questions with Cronbach's *⍺* was used. At the end, the data collected from 25 public health students were entered into SPSS.19 and calculated, Cronbach's *⍺* formula in this calculation was the following formula.

### 2.6 Data collection and data analysis

The questionnaires were electronically prepared, and the links to the questionnaires were sent via email to the experts. Due to the large number of questions and questionnaires, the tools were provided to experts gradually and with time intervals.

### 2.7 Data analysis

For determining the CVI and CVR, data analysis is descriptive based on content validity formulas, and in the case of reliability, in the case of knowledge questions, using the Kuder–Richardson formulas, and in the case of multivalued questionnaires, the Likert scale is based on the internal consistency of items method and Cronbach's *⍺* coefficient was used.

### 2.8 Ethical consideration

Written ethical approval was taken from the SMART University of Medical Sciences' local ethics committee (approval number (Ref.No. IR.VUMS.REC.1401.017)) and written informed consent obtained from all the participants.

## 3 RESULTS

Based on the results of this study, questionnaires to measure students' KPS from the educational technology course with 118 questions and in three areas were designed and validated with the help of scientific sources and experts' opinions.

### 3.1 Demographic characteristics

In the qualitative phase, the opinions of 10 experts specializing in medical education (3), health education (3), and educational technology (4) were used to measure content validity. Six of them were female and four were male, and their teaching experience was between 3 and 21 years.

In quantitative stage after confirming the content validity of tools, to determine the reliability, these tools were implemented on 50 public health students who took the educational technology course. Of 50 participants 44 (88%) students were female and 6 (12%) were male. Their age range was between 19 and 22 years. Their grade point average (GPA) was between 14 and 18 and none of them had taken this course before or participated in educational technology workshops.

### 3.2 Validation of knowledge assessment tools

Based on the results of the study, in the analysis of the CVI, the items that had CVR higher than 0.62, it was confirmed that the total CVR index was 0.885 and the total CVI was 0.862. Also, the value of CVI for relevance index (0.835), Clarity (0.863), and Simplicity (0.891) has been confirmed. The reliability of the questionnaire was 0.780 based on the Kuder–Richardson coefficient, which shows that all the knowledge questions (20 questions) have high reproducibility and were accepted (Table 2).

**Table 2:** Psychometric characteristics of each item in the knowledge assessment tools.

Components	Items	Content validity				Reliability
		CVR	CVI			High coefficient KR‐20
		Necessity	Simplicity	Clarity	Relevance	
*Principles of*:						
Nonreflective educational media	Q01	0.90	0.85	0.85	0.80	0.780
	Q02	0.90	0.80	0.85	0.80	
	Q03	0.85	0.80	0.85	0.85	
	Q04	0.83	0.80	0.80	0.85	
	Q05	1.00	0.85	0.80	0.90	
	Q06	0.85	0.90	0.80	0.80	
	Q07	0.85	0.90	1.00	0.80	
Reflective educational media	Q08	1.00	0.90	1.00	0.85	
	Q09	0.85	1.00	0.85	0.85	
	Q10	0.90	1.00	0.89	0.90	
	Q11	0.90	1.00	1.00	0.80	
	Q12	0.90	0.85	0.85	0.85	
	Q13	1.00	0.80	0.85	0.85	
	Q14	0.80	0.80	0.85	0.85	
Communication	Q15	0.88	0.85	0.85	0.90	
	Q16	0.85	0.85	0.85	0.80	
Role‐playing	Q17	0.80	0.90	0.80	0.80	
	Q18	0.99	0.98	0.81	0.85	
	Q19	0.80	0.98	0.81	0.80	
	Q20	0.85	0.99	0.90	0.80	
Total	–	CVR = 0.885	0.891	0.863	0.835	
			CVI total = 0.862			

### 3.3 Validation of performance assessment tools

Based on the study, a performance questionnaire with 80 questions was designed in four areas: poster, pamphlet, playing the role of a teacher in the training program and slides prepared in PowerPoint software. In the poster section, the CVR was 0.906 and the CVI index was 0.871 (Table 3). For pamphlet, CVR was 0.877 and the CVI index was 0.866 (Table 4). In the area of slides prepared in PowerPoint software, the total index of CVR was 0.960 and CVI was 0.981 (Table 5). Also in the part of role‐playing as a teacher, the total CVR index was 0.980 and CVI was 0.871 (Table 6). In checking the reliability of the questionnaire; Cronbach's *⍺* coefficient was evaluated for each of the performance subscales included: poster and pamphlet tools (*r* = 0.880), PowerPoint (*r* = 0.871), and role‐playing (*r* = 0.890), which shows that this instrument has high reliability (Tables 3, 4, 5, 6).

**Table 3:** Psychometric characteristics of each item in the performance assessment for Poster tool.

Components	Items	Content validity				Reliability	
		CVR	CVI			Cronbach's *⍺*	
		Necessity	Simplicity	Clarity	Relevance	Factors	If item deleted
Configuration	Appropriate title	0.80	0.85	0.85	0.85	0.895	0.81
	Introducing the teacher	1.00	0.80	0.85	0.80		0.81
	Introducing the Learners	0.70	0.80	0.85	1.00		0.81
	Title and logo of the center	1.00	0.90	0.80	1.00		0.81
	Date of preparation	0.80	0.90	0.80	1.00		0.81
	Contact us	0.80	0.90	0.80	0.80		0.80
Perspective	Simplicity	0.90	0.90	1.00	0.80	0.948	0.80
	Proper margining	1.00	0.90	1.00	0.85		0.81
	Decent visual quality	1.00	0.90	0.85	0.90		0.81
	Contrast (text and background)	0.90	0.90	0.90	0.90		0.81
Content	Appropriate content	1.00	0.90	1.00	0.80	0.793	0.81
	Logical sequence of content	1.00	0.85	0.85	0.85		0.81
	Up‐to‐date content	0.85	0.80	0.85	0.85		0.81
	Citing sources	0.85	0.80	0.85	0.85		0.81
Visual elements	Appropriate images/charts/tables	1.00	0.80	0.85	1.00	0.717	0.80
	Composition and arrangement	0.85	0.85	0.85	0.90		0.77
	Size of text	0.90	0.90	0.80	0.80		0.80
	Font of text	0.85	0.95	0.80	0.90		0.80
	length of sentences	0.90	0.95	0.80	0.80		0.80
	Line spacing	1.00	1.00	0.90	0.80		0.80
Total		CVR = 0.906	0.879	0.863	0.872	*R* = 0.880	
			CVI total = 0.871				

**Table 4:** Psychometric characteristics of each item in the performance assessment for Pamphlet tool.

Components	Items	Content validity				Reliability	
		CVR	CVI			Cronbach's *⍺*	
		Necessity	Simplicity	Clarity	Relevance	Factors	If item deleted
Configuration	Appropriate title	0.80	0.85	0.85	0.80	0.895	0.81
	Introducing the teacher	1.00	0.80	0.85	0.90		0.81
	Introducing the Learners	0.70	0.80	0.85	0.85		0.81
	Title and logo of the center	1.00	0.80	0.80	0.85		0.81
	Date of preparation	0.80	0.85	0.80	0.90		0.81
	Contact us	0.75	0.90	0.80	0.80		0.80
Perspective	Simplicity	1.00	0.90	1.00	0.80	0.948	0.80
	Proper margining	0.80	0.90	1.00	0.85		0.81
	Decent visual quality	0.90	1.00	0.85	0.85		0.81
	Contrast (text and background)	0.85	1.00	0.90	0.90		0.81
Content	Appropriate content	0.80	1.00	1.00	0.80	0.793	0.81
	Logical sequence of content	0.99	0.85	0.85	0.85		0.81
	Up‐to‐date content	0.85	0.80	0.85	0.85		0.81
	Citing sources	0.85	0.80	0.85	0.85		0.81
Visual elements	Appropriate images/charts/tables…	0.90	0.85	0.85	0.90	0.717	0.80
	Composition and arrangement	0.85	0.85	0.85	0.80		0.77
	Size of text	0.90	0.90	0.80	0.80		0.80
	Font of text	0.90	1.00	0.80	0.85		0.80
	length of sentences	0.90	1.00	0.80	0.80		0.80
	Line spacing	1.00	1.00	1.00	0.80		0.80
Total		CVR = 0.877	0.894	0.866	0.841	*R* = 0.880	
			CVI total = 0.866				

**Table 5:** Psychometric characteristics of each item in the performance assessment for PowerPoint tool.

Components	Items	Content validity				Reliability	
		CVR	CVI			Cronbach's *⍺*	
		Necessity	Simplicity	Clarity	Relevance	Factors	If item deleted
Configuration	Introduce title and teacher	1.00	1.00	1.00	1.00	0.770	0.80
	Learning objectives	0.80	0.90	1.00	1.00		0.85
	Provide the summary at the end	0.70	0.90	1.00	0.80		0.77
	Citing sources at the end	1.00	1.00	1.00	1.00		0.81
Arrangement of contents	Heading and sub‐heading	1.00	1.00	1.00	1.00	0.850	0.81
	Narrative and logical sequence	1.00	1.00	1.00	1.00		0.81
	Numbering the heading/sub‐heading	1.00	1.00	1.00	1.00		0.81
Scientific content	Knowledgeable content	1.00	1.00	1.00	1.00	0.850	0.80
	Up‐to‐date content	1.00	1.00	0.80	1.00		0.85
	Related and effective content	1.00	1.00	1.00	1.00		0.77
	clear	1.00	1.00	1.00	1.00		0.81
Adjust content	Font of text	1.00	1.00	1.00	1.00	0.730	0.81
	Size of text	0.70	1.00	1.00	0.90		0.81
	Coherence	1.00	0.80	1.00	1.00		0.81
	Segmentation	1.00	1.00	1.00	1.00		0.80
	Appropriate images/charts/tables	1.00	1.00	1.00	1.00		0.85
	Contrast (text and background)	1.00	1.00	1.00	1.00		0.77
	Appropriate animations	1.00	1.00	1.00	1.00		0.81
Emphasis and attention	Highlighting the main points	1.00	1.00	1.00	1.00	0.660	0.81
	Signaling the main points	1.00	1.00	1.00	1.00		0.81
Total		CVR = 0.960	0.980	0.991	0.985	*R* = 0.871	
			CVI total = 0.981				

**Table 6:** Psychometric characteristics of each item in the performance assessment for Role Play tool.

Components	Items	Content validity				Reliability	
		CVR	CVI			Cronbach's *⍺*	
		Necessity	Simplicity	Clarity	Relevance	Factors	If item deleted
Role mastery	Clarify the issue	1.00	0.85	0.85	0.85	0.791	0.85
	Content transfer	0.8	0.85	0.85	0.85		0.79
	Role‐playing power	0.9	1.00	1.00	0.90		0.81
	Emotion control	1.00	1.00	1.00	0.90		0.80
	Power of speech	1.00	1.00	0.90	1.00		0.80
	The right start	1.00	0.85	0.85	0.85		0.81
Design and Implementation	Important social goals	1.00	0.85	0.85	0.85	0.948	0.81
	Interesting subjects	1.00	0.90	0.90	0.90		0.80
	Execute and discuss	1.00	0.90	1.00	0.80		0.80
	Creating learners motivation	1.00	0.80	0.80	0.80		0.81
	Motivate students' participation	1.00	0.85	0.85	0.85		0.81
	Motivate students' kinetic skills	1.00	0.85	0.85	0.85		0.81
	Reinforce new values and attitudes	1.00	1.00	1.00	1.00		0.80
	Increase confidence in presentation	1.00	1.00	0.90	0.90		0.80
	Strengthen the expression of words	1.00	0.85	0.90	0.90		0.85
Control of conditions	Management and organization	1.00	0.85	0.85	0.85	0.884	0.77
	Determining roles	0.90	0.85	0.85	0.85		0.81
	Provision of facilities/equipment	1.00	1.00	0.90	1.00		0.81
	Right place	1.00	0.80	0.80	0.80		0.81
	Time	1.00	0.85	0.85	0.85		0.81
Total		0.980	0.895	0.887	0.832	*R* = 0.890	
		CVR = 0.980	CVI total = 0.871				

### 3.4 Validation of satisfaction assessment tool

Based on the findings of the study; The questionnaire of students' satisfaction with the quality of the course was designed with 18 questions. The CVI was 0.949, the CVR was 0.861, and the overall reliability was 0.928, which indicates the high reliability of this tool and can be used to determine satisfaction with the quality of the practical course (Table 7).

**Table 7:** Psychometric characteristics of each item in the satisfaction assessment tool.

Components	Items	Content validity				Reliability	
		CVR	CVI			Cronbach's *⍺*	
		Necessity	Simplicity	Clarity	Relevance	Factors	If item deleted
Motivation	This course motivated me to learn more	1.00	1.00	0.80	0.80	0.832	0.85
	This method encouraged me to participate more in my learning	0.80	1.00	0.80	0.80		0.79
Learning	This course increased my knowledge and I learned various things	0.80	1.00	1.00	1.00	0.737	0.81
	This method made me learn the material more deeply	0.70	1.00	0.80	0.80		0.80
	This method makes it possible to keep the contents and remember them more	0.70	0.90	1.00	1.00		0.80
Problem solving	This method makes me think and generate more ideas	1.00	1.00	0.80	0.80	0.758	0.81
	This course encouraged me to recognize, analyze, and solve problems	0.80	1.00	1.00	1.00		0.81
	This method strengthened my ability to reason, evaluate, and judge	1.00	1.00	1.00	1.00		0.81
Team activity	This method increases communication and team activities among students	1.00	1.00	1.00	1.00	0.735	0.80
	This method increases responsibility in group activities	0.80	1.00	1.00	0.90		0.81
	This method strengthens the ability to criticize and accept the opinions of others	0.80	1.00	1.00	1.00		0.81
Interaction	This method strengthens my ability to discuss and exchange opinions with others	0.80	1.00	1.00	0.90	0.728	0.80
	This method increases the student‐teacher interaction and receiving feedback	0.80	1.00	1.00	1.00		0.80
	This method provides free interactions with others and easy expression of opinions	0.80	1.00	1.00	1.00		0.80
Time Management	This method saves and optimally uses time	0.85	1.00	0.80	0.80	0.630	0.80
	This method strengthened my ability to manage time and schedule	0.85	1.00	1.00	1.00		0.80
Application	This method was effective in teaching the practical activities of the lesson	1.00	1.00	1.00	1.00	0.725	0.80
	This method made me better able to apply the theoretical material in practice	1.00	1.00	0.80	0.80		0.80
Total		CVR = 0.861	0.994	0.933	0.922	*R* = 0.928	
			CVI total = 0.949				

## 4 DISCUSSION

The purpose of the present study was to designing and validating questionnaires for evaluating the KPS of public health students in the practical course of educational technology. This study described the development and validation of tools based on face validity, content validity (CVI, CVR), and reliability (Internal consistency).

*Knowledge*: Based on the results of the study, the analysis of the CVI and CVR revealed important insights. Based on the Lawshe model if the experts are 10 members, items with a CVR higher than 0.62 confirmed.^39^, ^41^ In this research a total CVR index of 0.885 which shows the very good validity of the knowledge measurement tool. Also a total CVI of 0.862. Additionally, the CVI values for relevance (0.835), clarity (0.863), and simplicity (0.891) were confirmed, indicating strong content validity across these dimensions. In the examination of CVI, Waltz and Bausell stated that if the value of CVI is greater than 0.79, it is considered very good and acceptable. For values between 0.70 and 0.79, it is necessary for the items to be reviewed again or removed. In line with the CVR results, the CVI values also indicate a very good validity of the tool.^40^, ^41^ Moreover, the reliability of the knowledge assessment questionnaire, assessed using the Kuder–Richardson coefficient, was found to be 0.780, demonstrating high reproducibility and acceptance of all items. The Kuder–Richardson coefficient, specifically the Kuder–Richardson Formula 20 (KR‐20), is a measure of internal consistency reliability used for tests with dichotomous choices, where each question has two possible answers (right or wrong). This coefficient assesses how consistent the results from the test are and how well the test measures what it is intended to measure. The KR‐20 formula calculates the reliability of a test by considering the total number of questions, the proportion of individuals who answered each question correctly, the proportion of individuals who answered each question incorrectly, and the variance of scores for all individuals who took the test^42^, ^43^ The KR‐20 value ranges from 0 to 1, with higher values indicating higher reliability. Typically, values above 0.70 are considered acceptable, while values above 0.90 suggest a homogeneous test. According to the reliability value of the knowledge measurement tool in this research (0.780), the reliability of this tool is reported as good.

*Performance*: In the evaluation of student performance using assessment tools for designing and producing PowerPoint, posters, pamphlets, and role‐play tools, the CVR was examined. The CVR values for the checklist of posters (0.906), pamphlets (0.877), PowerPoint (0.960), and role‐play tools (0.980) were confirmed. With 10 experts involved, a minimum agreement score of 0.62 was expected. The findings revealed excellent content validity scores for all tools based on the CVR.^39^, ^41^ Moreover, the CVI scores exceeding 0.890 for all tools indicate high validity according to Waltz and Bausell's model.^40^

The reliability of performance questionnaires with 80 items in four assessment tools was confirmed using Cronbach's *⍺* values: Poster and pamphlet (0.880), Powerpoint (0.871), and role‐playing (0.890). Reliability coefficients typically range from 0 to 1, with values above 0.90 indicating excellent reliability, values between 0.80 and 0.90 considered very good for classroom tests, and values between 0.70 and 0.80 considered good for classroom tests.^44^, ^45^ Based on research findings, all fundamental values for performance measurement tools have been reported to be over 0.870, indicating that the designed tools exhibit excellent reliability.

Although we did not find exactly the same and articles research about knowledge and performance assessment to compare with the current research, our review of studies dealing with psychometric instruments revealed that the CVI and CVR indices, along with Cronbach's *⍺* reliability, are commonly utilized in most of these studies.^46^, ^47^, ^48^ Goni et al., in a study titled “Design and Validation of Knowledge, Attitude and Performance Questionnaire in relation to infection prevention” designed a tool for this purpose. The results of the study indicated satisfactory psychometrics for measuring knowledge and the reliability coefficient of knowledge was 0.92.^49^ In the study of Rezaei et al., the CVI was equal to 0.922 and Cronbach's *⍺* coefficient was equal to 0.784, which was consistent with the results of our study.^50^ In the study of Marashi et al. regarding the design of the health faculty students' awareness questionnaire in the field of AIDS, the reliability of the tool was 0.79, which is in line with this study.^51^ In a research conducted by Nasiri et al.^52^ in their study regarding the performance of contractors in the petrochemical industry, they designed a performance questionnaire, which included 7 indicators with 35 criteria. To check the validity and reliability of this tool, the opinions of six university professors were used. Based on the results, CVR, CVI, and Cronbach's *⍺* coefficient are 0.54, 0.728, and 0.782, respectively, which CVR, CVI, and Cronbach's *⍺* coefficient were higher in our research. In the study of Mohammad Abadi et al.^53^ regarding the design and validation of the performance questionnaire of knowledge‐based companies, with 17 statements in two dimensions, the reliability criterion of the entire questionnaire was 0.89 and it had good validity and reliability, which is consistent with the results of the present study. Also, the study of Ahanchian^54^ in relation to the validation of the performance evaluation questionnaire is consistent with the results of the present study.

*Satisfaction*: Based on the results of the present study, in the satisfaction section, 18 questions in seven areas with a 5‐option Likert scale were designed in the field of motivation, learning, problem solving, team activity, interaction, time, and application, which actually express the satisfaction of students with the quality of the course. It is practical. Based on the findings of the present study, the course satisfaction questionnaire had a high CVI (0.949) and CVR (0.861) and a high overall reliability of 0.928. In addition to the total reliability, the reliability of each of the components has also been evaluated at a favorable level (0.66 to 0.830). It is worth mentioning that the reliability is greatly affected by the sample size. Although the number of samples was 50 people, the reliability value of the components is acceptable and suitable.

In assessing questionnaire reliability, it is important to consider the impact of individual items on the overall reliability of the scale. The “If item deleted” analysis provides insights into how removing specific items affects the reliability of the questionnaire. By analyzing statistics such as average scale values, variance, correlation coefficients, and Cronbach's *⍺* after deleting an item, researchers can determine if removing certain items would enhance the overall reliability of the questionnaire. Obviously, if by removing an item, the reliability value of the items decreases, it indicates that that item had a positive effect on the reliability. As in all research tools, including satisfaction measurement, it shows that the impact of all items on total reliability was positive.^55^ Various tools have been designed to measure students' satisfaction with the educational environment. The research of Madvari^56^ was conducted in relation to the validity and reliability of the employee satisfaction questionnaire, the questionnaire designed for employee satisfaction was higher than the standard in terms of CVI (0.79) and CVR (0.55). The *⍺* coefficient obtained showed the high internal consistency of the questionnaire, also in the study of Torkzadeh et al.^35^ the academic satisfaction scale of students had high validity and reliability. In the study of Satayshi et al.,^36^ the reliability of the Lent academic satisfaction scale in Iranian students was at a favorable level and was 0.81, which was consistent with the results of our study. In the study of Montejano‐Lozoya^57^ regarding the design and validation of the questionnaire for measuring satisfaction with the practical training of nursing students, the validity and high Cronbach's *⍺* value (0.91) showed that all the items have high reliability and according to the results; It is a reliable tool to measure nursing students' satisfaction with practical training in clinical settings.

### 4.1 Strengths and limitations

This research used new tools in the targeted training and evaluation of students in the course of practical educational technology, and the psychometric results also indicate the validity of this tool. But it should be noted that this tool is being used for the first time and it is better to implement and check and retest it in different environments. It is important to clarify that this study focused solely on public health students in a single course, cautioning against broad generalizations of the findings. It is important to note that the study was conducted in a small area with a limited population. (Each semester, 25 students participate in this course and We included two groups who had completed the course in consecutive semesters to evaluate the questionnaire's reliability). Nevertheless, it is clear that this study should be replicated and tested with a larger sample size. In addition, to assess the long‐term impact of these educational tools on learning, longitudinal studies are necessary). Furthermore, the predominant gender ratio among students was female, suggesting potential variations in settings with diverse demographic factors. Moreover, the course content could be influenced by the specific curriculum of the university, indicating a need for tailored checklists based on varying lesson structures.

### 4.2 Suggestions for future studies

The validity of the tool in this study was assessed through face and content validity. It is anticipated that the construct validity will be examined using factor analysis with a large sample size. Furthermore, it is recommended that the tools developed in this study be utilized in educational intervention research. Another important aspect is that the utility of the instruments developed in this study extends beyond health students. Given that experts in education and health have validated these tools, they can be applied to assess the effectiveness of various educational materials like posters, pamphlets, role‐playing, and PowerPoint presentations across diverse educational settings. Future studies could explore the validity and reliability of these tools further, as well as compare the knowledge and skills of learners utilizing these tools in various educational contexts.

## 5 CONCLUSIONS

Practical and experiential learning is less structured and more reliant on opportunities compared with purely theoretical education. As a result, it may sometimes receive less attention or not be evaluated with concrete measures. For instance, the course on educational technology in the public health field is a crucial practical component that effectively conveys educational concepts to students. Developing appropriate tools for structured education is essential. Beyond the realm of public health, individuals in education, including professors and students, utilize content development tools such as PowerPoint, pamphlets, posters, infographics, and more. The validated tools from this research can be used for assessing content across various educational contexts.

Furthermore, when quality indicators for practical training are established beforehand, they can serve as a guide for students and make their educational expectations clearer.

Although various tools exist for determining the quality of educational content, the tools developed in this research are tailored to achieve educational goals and are particularly suitable for various types of educational content, incorporating the perspectives of educational experts, health professionals, and experienced tool developers.

Additionally, the tool's credibility has been confirmed by participants' feedback during the course (students), making it potentially applicable in similar fields. In particular, with the emergence of new technologies, the field of educational content production has expanded, and there is a need to develop tools for assessing the quality of this content. However, since new technologies are constantly evolving, and with the emergence of artificial intelligence and machine learning, new types of educational content and materials are gaining attention, these tools and indicators must undergo continuous updates.

It is important to note that the findings of this research provide a set of common primary tools that are typically taught in technology education courses, but due to the limited sample size, it was not possible to establish construct validity, and it is necessary to conduct a comprehensive exploratory and confirmatory analysis to ensure the composition of the tools in in forthcoming studies.

## AUTHOR CONTRIBUTIONS

**Fatemeh Darabi**: Conceptualization; investigation; writing—original draft; validation; writing—review and editing; data curation; funding acquisition; methodology. **Zahra Karimian**: Conceptualization; investigation; writing—original draft; methodology; validation; writing—review and editing; formal analysis; project administration; supervision; data curation; visualization.

## CONFLICT OF INTEREST STATEMENT

The authors declare no conflict of interest.

## ETHICS STATEMENT

This research is a part of Dr. Fatemeh Darabi's master's thesis in medical education, which was approved after receiving approval from the Medical Ethics Committee of the Research Vice‐Chancellor of Smart University of Medical Sciences (project code 394 and project ethics code number IR. VUMS.REC.1401.017) has been approved. All participants in the research were fully informed about the objectives of the study and provided their informed consent before participating. Confidentiality of the information was assured to all participants. The questionnaires were collected and analyzed anonymously to protect the privacy of the respondents. The final report of the research was shared with the relevant authorities for future planning purposes.

## TRANSPARENCY STATEMENT

The lead author Zahra Karimian affirms that this manuscript is an honest, accurate, and transparent account of the study being reported; that no important aspects of the study have been omitted; and that any discrepancies from the study as planned (and, if relevant, registered) have been explained.

## Data Availability

The data sets used and/or analyzed during the current study are available from the corresponding author on reasonable request. The corresponding author had full access to all of the data in this study and takes complete responsibility for the integrity of the data and the accuracy of the data analysis.
